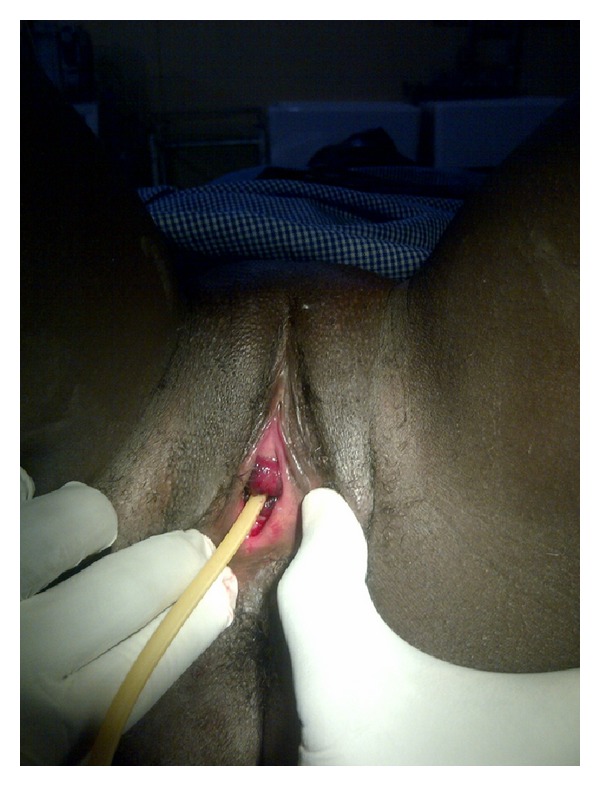# Urethral Mucosa Prolapse in an 18-Year-Old Adolescent 

**DOI:** 10.1155/2013/231709

**Published:** 2013-09-17

**Authors:** Akadiri Olumide, Ajenifuja Kayode Olusegun, Bakare Babatola

**Affiliations:** ^1^Department of Obstetrics and Gynaecology State Specialist Hospital Ondo, Ondo State, Nigeria; ^2^Department of Obstetrics, Gynaecology & Perinatology, Obafemi Awolowo University/Teaching Hospital, Complex, Ile-Ife, Osun State, Nigeria

## Abstract

Urethra mucosa prolapse is a benign condition in which there is a circular protrusion of the distal urethra through the external urethra meatus. It is more commonly seen in prepubertal black girls and postmenopausal white women. It is rare in the reproductive age group. This case describes the presentation and management of an 18-year-old adolescent with urethra mucosa prolapse.

## 1. Introduction

Urethral mucosa prolapse is a benign condition which was first described in 1732 by Solingen [1]. It is a condition in which there is a circular protrusion of the distal urethra through the external urethra meatus. The protruding mass is tubular and covered by easily bleeding mucous membranes. It is a rare condition and it is most commonly seen in prepubertal black girls between the ages of 6 months and 8 years and postmenopausal white women. The majority of cases (80%) are in the paediatric age group and incidence in this age group is 1 : 3000 [2]. In the postmenopausal age group, white women are most commonly affected in approximately 86% of cases [2].

Estrogen deficiency has been associated. It may be complicated by strangulation.

Urethra mucosa prolapse is a rare condition and even more rare in the reproductive age group. We present this case of urethra mucosa prolapse in an 18 year old adolescent.

## 2. Case Report

Miss U. A. is an 18-year-old student of a tertiary institution. Her last menstrual period was 10 days prior to presentation which was normal in quantity and duration. She presented in the gynaecological emergency unit of the State Specialist Hospital Ondo with a history of bleeding per vaginam of about 2 days duration prior to presentation. Bleeding was associated with passage of clots. There was no associated abdominal pain, and she is not sexually active. There was no history of vagina trauma or instrumentation. She attained menarche at the age of 14 years and menstruates for 3 days in a cycle of 26–28 days.

 Examination revealed a healthy looking but anxious young female. She was not pale, anicteric, and afebrile. Vital signs revealed temp = 36.8°C, pulse rate = 100 beats/min, respiratory rate = 18 cycles/min and blood pressure = 120/80 mmHg.

**Figure 1:**
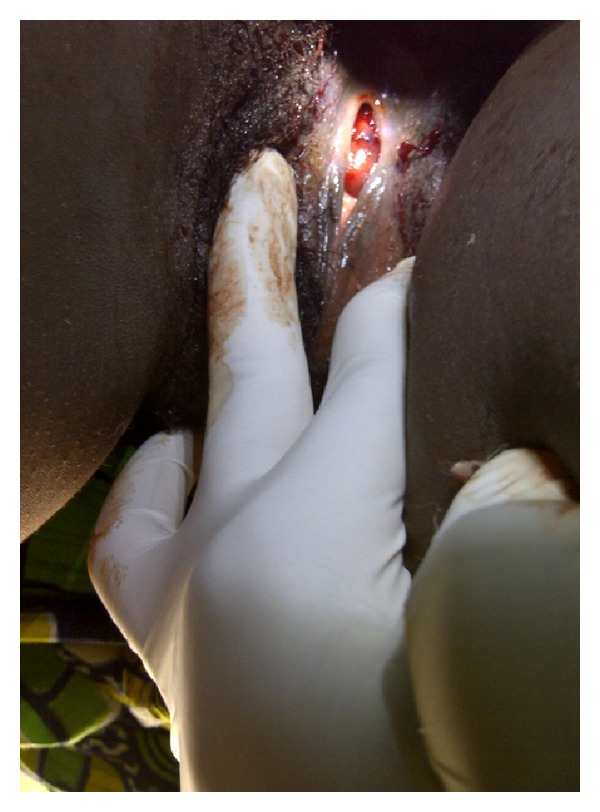


There was no tenderness on abdominal examination and no palpably enlarged organ. Vagina examination revealed intact hymen. There was a polypoid hyperemic mass with a central dimple actively bleeding with some clots on it (Figure 1). The periurethral vaginal mucosa was normal.

An assessment of urethra mucosa prolapse was made which was confirmed by passing a size 16 F urethra catheter through the dimple (Figure 2). Investigations done included an FBC and urine m/c/s which did not yield any growth. She was reassured and counselled for surgical excision, and consent was obtained. This was done under general anaesthesia using the modified Kelly-Burnham procedure. Chromic catgut 3/0 was placed in four quadrants of the prolapsed mucosa with a urethral catheter in situ. The prolapsed urethra mucosa was carefully excised, and the urethra mucosa was transfixed using the chromic catgut in the four quadrants. The catheter was left in situ for 48 hrs, and after removal, she voided freely without any difficulty. The excised prolapsed tissue was sent for histopathology which confirmed it was consistent with urethral mucosa. She was discharged on third day postoperatively and she has been on followup for six months with no complication.

## 3. Discussion

Urethral mucosa is a rare condition. It is seen more in prepubertal girls and postmenopausal white women [3]. This is to our knowledge the first reported case in an adolescent. The exact cause of urethra prolapse is not known, but multiple aetiologies have been proposed including increased urethra mobility, mucosal redundancy, increased abdominal pressure, and poor attachment between the muscular layers of the urethra [4]. Other contributing factors include estrogen deficiency and poor nutrition and hygiene [5, 6].

The urethra is composed of inner longitudinal and outer circular-oblique smooth muscle layers. Usually, a natural cleavage plane is present between the inner and outer muscle layers. A prolapsed urethra may result from these 2 muscle layers separating after a sudden episodic increase in intraabdominal pressure. Disruption of these muscle layers results in complete and circular eversion of the urethral mucosa through the external meatus and leads to urethral prolapse. Swelling and congestion of the prolapsed mucosa create a purse-string effect around the distal urethra, impeding venous return and exacerbating vascular congestion. If left untreated, urethral prolapse may progress to strangulation and eventual necrosis of the protruding tissues. The fundamental anatomical defect of urethral prolapse is the separation of the longitudinal and circular-oblique smooth muscle layers [7]. Surgical apposition of these smooth muscle layers is curative [1]. 

Prepubertal urethral mucosa prolapse is predominantly asymptomatic. Often it is an incidental finding during routine examination. The most common presentation is vaginal bleeding associated with a periurethral mass. Until urethral prolapse is definitively diagnosed, the presence of blood in the genital area should raise the suspicion of sexual abuse in this age group. In rare circumstances, children may present with voiding disturbances such as dysuria, frequency, introital pain, or haematuria.

Patients with postmenopausal urethral prolapse are often symptomatic. Vaginal bleeding associated with voiding symptoms is fairly common. If the prolapsed urethra is large, the mucosal mass may become strangulated, which results in venous obstruction, thrombosis, and necrosis of the prolapsed tissue.

Upon physical examination, urethral prolapse appears as a doughnut-shaped mass protruding from the anterior vaginal wall. The mass may be painful and tender to palpation. The mucosa is ulcerated in most cases and usually bleeds upon contact. The congested mucosa may appear bright red or dark and cyanotic.

Diagnosis of urethra mucosa prolapsed is clinical and is made by visualization and catheterization of the central opening within the prolapsed urethra mucosa (Figure 2). Extensive investigations is not necessary to make the diagnosis; however, ultrasound can be done to exclude malignancy and other abnormalities of the kidneys and bladder [7, 8]. A very close differential is urethra caruncle which is described as a fleshy outgrowth of the distal mucosa usually originating from the posterior lip; however, urethral caruncles are focal, while prolapse are circumferential. It might also be confused with sexual abuse. Other differential diagnoses include urethral or vaginal malignancy, condyloma, and rhabdomyosarcoma.

Treatment can be either surgical or medical. Medical treatment includes local hygiene, sitz bath, topical antibiotics, steroid, or estrogen creams [1]. Most of the success with the nonsurgical treatment is with the topical application of estrogen cream. Surgical intervention is indicated for more severe cases such as those with significant bleeding, thrombosis, or gangrenous changes and if use of estrogen cream is contraindicated [2]. Surgical treatment is best with the modified Kelly-Burnam operation in which the prolapsed mucosa is excised, and the mucocutaneous junction is reapproximated with absorbable sutures. Other surgical methods include keefe vagina/urethra placation, surgical reduction maintained with mattress sutures, manual reduction, and cautery excision [8]. The first line of treatment is medical; however, if medical therapy does not reduce the prolapse, then surgery is the treatment of choice. Medical management is not always an effective treatment for correcting prolapsed and may have a recurrence rate of 67%; surgery has a higher cure rate and relieves symptoms quickly [9–11]. Complications which may arise from surgical excision include urethral stenosis, urinary incontinence, and recurrence of the prolapse [5]. This patient had surgical treatment because of the severity of the bleeding.

Urethra mucosa prolapse is a rare condition. It is primarily associated with prepubertal girls and postmenopausal women. This case has shown that it can occur even in the reproductive age group. A high index of suspicion and careful clinical examination can help identify some of the cases within this age group especially when girls present with unexplained genital bleeding.

## Figures and Tables

**Figure 2:**